# Deep venous thrombosis in a 41-year-old female with May–Thurner syndrome following abdominoplasty and medial thigh lift

**DOI:** 10.1093/jscr/rjac175

**Published:** 2022-04-18

**Authors:** Joao Bombardelli, Jordan Kaplan, Andres F Doval, Norman H Rappaport

**Affiliations:** Department of Surgery, Houston Methodist Hospital, Weill Cornell Medicine, Houston, TX, USA; Department of Surgery, Division of Plastic Surgery, Baylor College of Medicine, Houston, TX, USA; Department of Surgery, Division of Plastic Surgery – Institute for Reconstructive Surgery, Houston Methodist Hospital, Weill Cornell Medicine, Houston, TX, USA; Department of Surgery, Division of Plastic Surgery, Baylor College of Medicine, Houston, TX, USA; Houston Center for Plastic Surgery, Houston, TX, USA

## Abstract

Deep venous thrombosis (DVT) is a feared occurrence following body contouring surgery as it can result in pulmonary embolism. Acute presentation can range from lower extremity edema and pain to being totally asymptomatic. Surgical literature reports reveal many risk factors for developing DVT, and surgeons must risk stratify their patients to best prevent this outcome. However, there are conditions which place patients at risk that are difficult to account for when making such decisions as they can be undiagnosed and are not a part of standard screening protocols. We present a case of DVT in a 41-year-old female with undiagnosed May–Thurner syndrome following abdominoplasty and medial thigh lift for massive weight loss. The authors discuss the current literature as well as challenges faced by surgeons who strive to appropriately risk stratify their cosmetic surgery patients to avoid complications such as venous thromboembolism.

## INTRODUCTION

Proximal lower extremity deep venous thrombosis (DVT) presents with venous thrombus of the popliteal (PPV), femoral (FV) or iliac veins. Over 90% of acute pulmonary embolism (PE) arise from these veins [1]. According to the Centers for Disease Control and Prevention, it is estimated that as many as 900 000 Americans suffer a venous thromboembolism (VTE) yearly with an estimation of 60 000 to 100 000 deaths [2]. In plastic surgery, the incidence of DVT is relatively low, 0.09% in a prospective cohort study done by Winocour *et al.* However, when combined procedures as well as procedures involving the trunk and extremities are performed, the risk of VTE increases significantly [3].

DVT should be suspected in patients who present with leg swelling, pain, warmth and erythema [4]. The pathology is ultimately driven by Virchow’s Triad of hypercoagulability, stasis and endothelial injury [5]. Multiple systems have been developed to predict and appropriately determine which patients would benefit from prophylactic measures. Currently, the Caprini Score is considered the most recognized and utilized [6]. Overtime the score has been modified to improve its efficacy [7].

The authors describe a case of DVT following abdominoplasty and medial thigh lift in a massive weight loss patient. The patient was found to have May–Thurner Syndrome (MTS), a high-risk diagnosis otherwise missed by conventional DVT risk stratification tools.

## CASE REPORT

A 41-year-old female underwent fleur-de-lis abdominoplasty and medial thigh lift. She did not have any significant medical history. She was ambulatory on postoperative day (POD) 0 and discharged the next morning. No chemoprophylaxis was given. On POD 11, she presented with left lower extremity (LE) swelling, discoloration and pain. Venous duplex study revealed a non-compressible DVT of the common femoral vein (CFV), FV, profunda vein, PPV, and posterior tibial veins.

**Figure 1:**
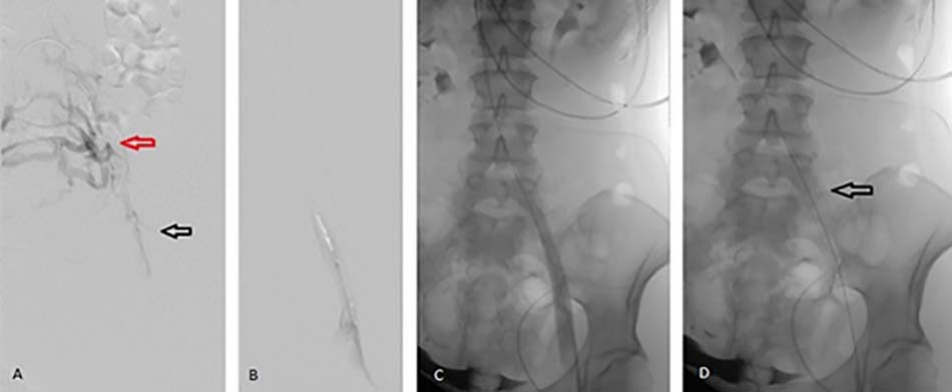
Angiographic images during percutaneous angioplasty of the left CIV. (**A**) Black arrow demonstrates left CIV stenosis secondary to anatomic compression with extensive clots. Red arrow demonstrates the collateral venous circulation, indicating chronicity of MTS. (**B**) Balloon angioplasty of the left CIV. (**C**) Left CIV patent post angioplasty. (**D**) Arrow demonstrates 16 × 60 mm stent deployed across the left CIV.

The patient underwent percutaneous thrombectomy of the extensive clot. Intravenous ultrasound (IVUS) revealed stenosis of the left common iliac vein (CIV) and multiple collaterals, indicating chronicity of the condition and compatible with MTS. Balloon angioplasty of the stenotic segment was performed and a 16 × 60 mm stent deployed. Repeat IVUS confirmed a patent stent and completion venogram showed good flow through the FV, CFV, external iliac vein (EIV) and CIV (Fig. 1).

The patient recovered well and was discharged on POD 2. She was placed on oral aspirin and apixaban. Six weeks post thrombectomy, in-office venous duplex ultrasound confirmed no evidence of DVT.

## DISCUSSION

Caprini *et al.* published the Clinical Assessment of Venous Thromboembolic Risk in Surgical Patients in 1991 after studying a group of 538 surgical patients. Patients were prospectively assessed based on a scoring system containing 20 risk factors, such as advanced age, previous history of VTE and present cancer. Patients were grouped into low, moderate and high risk for VTE. The study ultimately produced the Caprini Score for VTE [6]. Plastic surgery patients were not included in the initial cohort so further research has been done to validate the Caprini risk score for use in plastic and reconstructive surgery [7]. In addition, multiple studies have demonstrated that plastic surgery patients with higher Caprini scores are more likely to suffer a VTE event [8, 9].

According to the American Society of Plastic Surgeons, at least 18 000 annual cases of DVT occur between all plastic surgery cases across the United States [10]. Recent reports have discussed that the Caprini risk score fails to address how different plastic surgery procedures may affect the risk of developing a DVT. Winocour *et al.* were able to label patients undergoing body contouring procedures or combined aesthetic procedures as having an increased risk of developing a DVT regardless of surgery duration exceeding 6 h [3].

Swanson *et al.* recently evaluated general endotracheal anesthesia (GETA) as one of the potentially avoidable risks factors for VTE in plastic surgery. Patients that have surgery under monitored anesthesia care and/or epidural anesthesia have faster recovery and start mobilizing earlier when compared with those that undergo general anesthesia [11, 12].

Long haul travel (airline or car travel greater than 4 h) immediately following surgery increases the risk of DVT. Although pharmacologic prophylaxis would help prevent these episodes, further research is required to list specific recommendations for patients and physicians to follow [12, 13].

Rohrich *et al.* examined how standard tools such as the Caprini scale fail to fully address unique circumstances in plastic surgery, and furthermore in aesthetic surgery. Although many key risk factors for developing a DVT are included in the Caprini scale, the editorial cites the type of procedure performed, combining procedures, prolonged travel greater than 4 h immediately after surgery and the protective effect of not using GETA as key limitations to the scoring system [12].

For those whose Caprini risk score is ≥7, chemical prophylaxis is generally indicated. Low molecular weight heparin is preferred for those that have CrCl ≥30 ml/min and has a smaller risk of heparin-induced thrombocytopenia when compared with unfractionated heparin [12, 14].

DVT is a serious medical diagnosis. Patients who avoid progression to VTE and resultant PE may still suffer from a myriad of symptoms associated with post-thrombotic disorder. Prevention of this condition must remain at the forefront of clinical discussion as reactive treatment is not without consequences.

## CONFLICT OF INTEREST STATEMENT

None declared.

## FUNDING

None.
